# Advancing Nipple Reconstruction in Skin-Sparing Mastectomy: The Efficacy of the Long V-Y Flap Technique for Enhanced Size Retention and Symmetry

**DOI:** 10.3390/life16010088

**Published:** 2026-01-07

**Authors:** Woo Seob Kim, Byung Woo Yoo, Kap Sung Oh, Hyun Woo Shin, Kyu Nam Kim, Junekyu Kim

**Affiliations:** Department of Plastic and Reconstructive Surgery, Kangbuk Samsung Hospital, School of Medicine, Sungkyunkwan University, Seoul 03181, Republic of Korea

**Keywords:** nipple reconstruction, skin-sparing mastectomy, long V-Y flap technique, enhanced size retention, symmetry, split transplantation

## Abstract

Reconstruction of the nipple is the final step of breast reconstruction following skin-sparing mastectomy (SSM) and plays a critical role in restoring breast symmetry and aesthetic completeness. Various nipple reconstruction techniques have been described, including split transplantation of normal nipples and flap-based procedures; however, long-term maintenance of nipple projection and volume remains challenging due to postoperative tissue loss. This study evaluates the clinical outcomes of a previously described long V-Y flap technique, focusing on its ability to mitigate postoperative nipple projection and volume loss. A retrospective analysis was performed on 27 patients who underwent nipple reconstruction using the long V-Y flap following SSM with tissue expander–implant reconstruction. Nipple projection and volume were measured immediately after surgery and at follow-up beyond six months, and volume loss rates were calculated. Outcomes were compared descriptively with projection loss rates reported for other commonly used flap techniques in the literature. The mean nipple volume loss was 34.23%, and the mean projection loss was 32.79%, while nipple width remained largely unchanged over time. These values were numerically lower than those reported for skate, star, bell, and arrow flaps in prior studies. The long V-Y flap appears to be a practical and reliable option for nipple reconstruction after SSM, particularly for larger nipples, with favorable short- to mid-term maintenance of projection and volume.

## 1. Introduction

Skin-sparing mastectomy (SSM) is commonly performed for patients with nipple metastasis from breast cancer. Due to limited skin availability, most patients undergo tissue expansion and implant replacement. Nipple reconstruction is essential for improving breast completeness and achieving symmetry, representing the final step of breast reconstruction. Techniques for nipple reconstruction have been comprehensively categorized, and evidence-based decision-making algorithms have been proposed to guide technique selection based on patient factors, surgical timing, and breast reconstruction characteristics. Nevertheless, no single approach has consistently achieved durable long-term projection. Nipple reconstruction methods are broadly divided into split transplantation of the normal nipple and flap-based techniques [1,2,3,4,5,6]. Despite the use of various flap designs, long-term outcomes often remain suboptimal due to progressive projection and volume loss. Reported six-month projection loss rates across commonly used nipple reconstruction techniques range from approximately 30% for skate and modified star flaps to over 60% for bell flaps, underscoring the challenge of long-term projection maintenance [7,8,9,10].

This study aimed to calculate the long-term volume loss rate of reconstructed nipples, excluding cases of incomplete reconstruction due to flap necrosis or related complications. We analyzed contributing factors to nipple volume loss, explored mitigation strategies, and introduced the long V-Y flap as a streamlined and effective technique for nipple reconstruction [1]. This study offers a systematic evaluation of long-term outcomes of nipple reconstruction using this method, with comparison to other techniques reported in the literature. Despite previous publications describing the long V-Y technique, its long-term volumetric outcomes have not been consistently quantified using standardized measurements, nor sufficiently compared with other established nipple reconstruction methods.

## 2. Materials and Methods

### 2.1. Subjects

This study evaluated patients who underwent nipple reconstruction using the long V-Y flap technique after breast reconstruction with tissue expanders and prostheses following SSM between January 2016 and December 2022. Demographics and patient characteristics are listed in Table 1. Written informed consent was obtained from all 27 patients who participated in the study. The eligibility criterion for participation was a successful reconstruction without complications that could lead to incomplete nipple reconstruction. Patients with comorbidities known to impair wound healing, such as diabetes mellitus, vasculopathies or other vascular conditions were excluded to ensure an accurate assessment of the effectiveness of the long V-Y flap technique and to minimize confounding factors in nipple reconstruction outcomes. The study was conducted in accordance with the guidelines of the Declaration of Helsinki and was approved by the Institutional Review Board of Kangbuk Samsung Hospital (IRB no. KBSMC 2023-12-029; date of approval: 15 December 2023).

### 2.2. Operation

**Figure 1:**
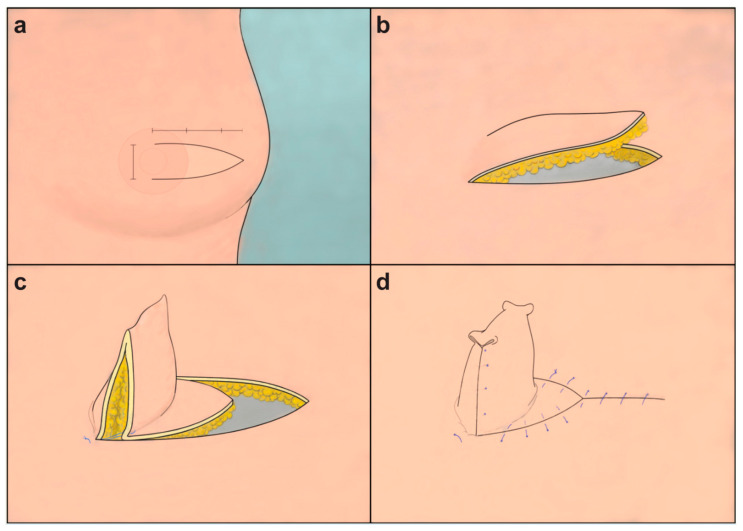
Diagrammatic representation of the long V-Y flap design for nipple reconstruction. (**a**) The flap base is marked at the planned nipple site, designed with a 1:2.5 width-to-length ratio and a half-fusiform shape, tapering in the middle. (**b**) The flap is meticulously separated with concurrent elevation of the skin and subcutaneous fat layers. (**c**) The flap is advanced toward the base and folded to achieve the desired height. (**d**) Depiction of the flap-folding, highlighting the inward manipulation of sharp rectangular tips at the top using a three-point suture, avoiding cuts that could restrict blood flow.

A centerline was drawn on the patient’s torso in a standing position, and the position of the nipple to be reconstructed was determined and marked on the opposite side of the normal nipple. A V-shaped flap was prepared using this point as the base of the long V-Y flap. In case of an existing scar, a flap was created to include the scar on one side; if the flap was distant from the existing scar, it was designed parallel to the scar. The flap width was 130% of the diameter of the normal nipple or approximately 3–5 mm wider. The flap was designed to be longer than wider with a width-to-length ratio of 1:2.5. Instead of a complete triangle, the shape of the flap was more effectively tapered, maintaining an appropriate width over the middle, shaped like half of a fusiform or wine glass. While separating the flap from the pectoralis major muscle or acellular dermal matrix (ADM), the skin and subcutaneous fat layers were raised together. By advancing the flap toward the base, it was folded to achieve the required height, and the triangular tip was positioned between the original base and the tip, where it was sutured in a Y-shape. If the apex of the nipple, where the flap folds sharply, is excised into a rounded shape, it could constrict the central portion of the flap, potentially compromising blood flow. Therefore, upper nipple resection is not recommended. To ensure unimpeded blood circulation to the distal portion of the flap, the apex was folded inwards and sutured to create a round contour (Figure 1). To prevent compression due to external forces, including the underwear, a hole was drilled in the center of the foam dressing to maintain and protect the height, and the sutures were removed approximately two weeks later.

### 2.3. Measurement of Nipple Projection and Volume Loss

**Figure 2:**
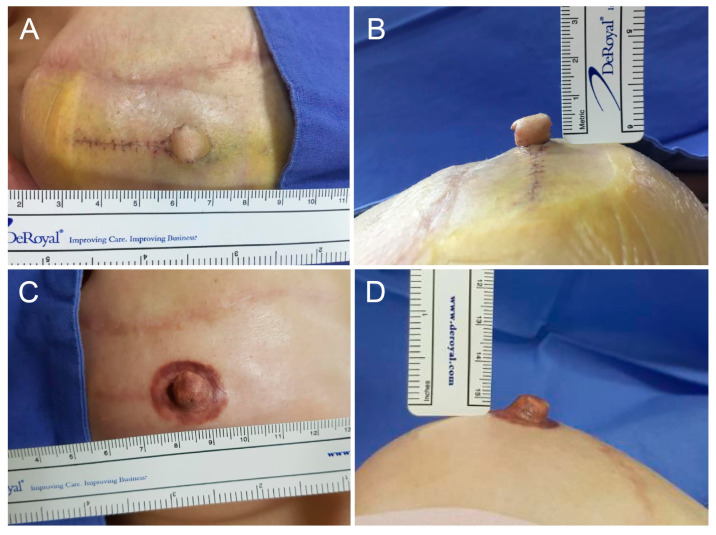
Clinical photographs of a 36-year-old female patient who underwent nipple reconstruction using the Long V-Y Flap Technique. (**A**,**B**) Immediate postoperative appearance showing a nipple width of 11 mm and height of 8 mm. (**C**,**D**) At 3 months postoperatively, the nipple maintained its width at 11 mm with a slight reduction in height to 7 mm.

**Figure 3:**
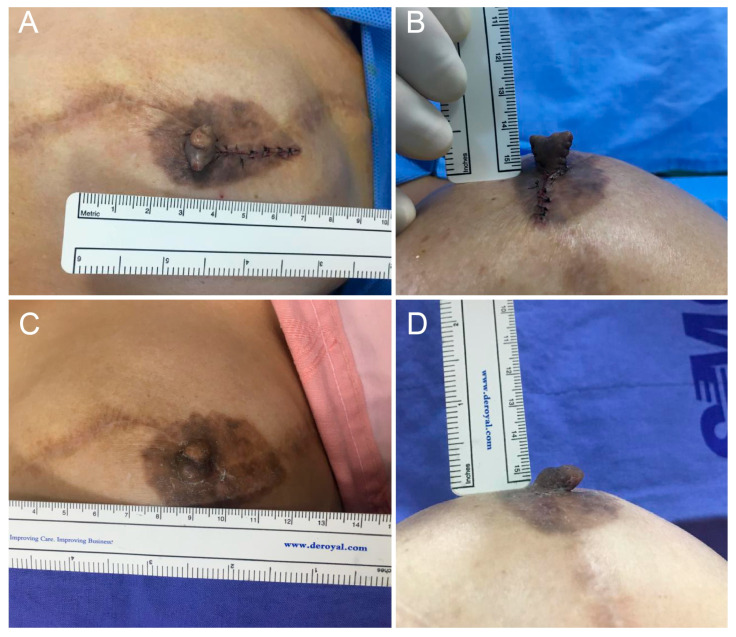
Clinical photographs of a 60-year-old female patient who underwent nipple reconstruction using the Long V-Y Flap Technique. (**A**,**B**) Immediate postoperative views showing a nipple width of 10 mm and height of 11 mm. (**C**,**D**) At 3 months postoperatively, the nipple width was slightly increased to 11 mm while the height decreased to 7 mm.

**Figure 4:**
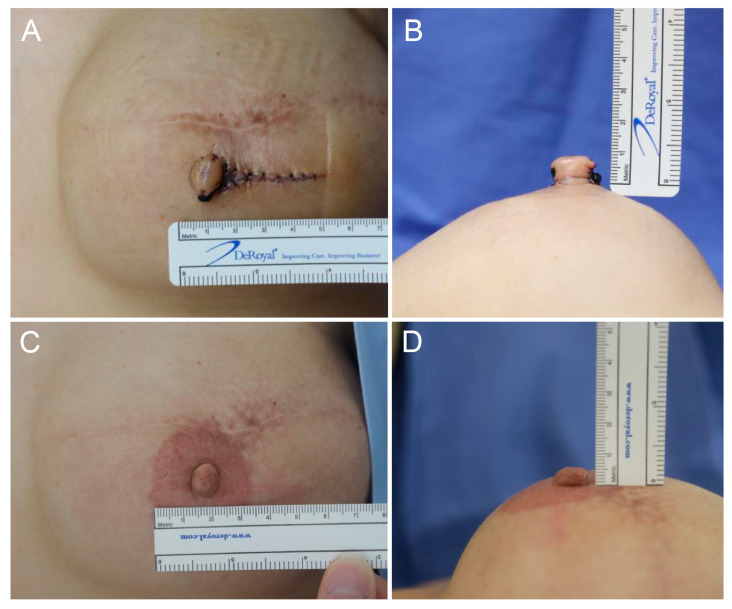
Clinical photographs of a 46-year-old female patient who underwent nipple reconstruction using the Long V-Y Flap Technique. (**A**,**B**) Immediate postoperative views demonstrating a nipple width and height of 8 mm. (**C**,**D**) At 6 months postoperatively, the nipple width had increased slightly to 9 mm while the height decreased to 6 mm.

In this study, volume retention (also referred to as persistence) is defined as the proportion of the initial nipple volume maintained at follow-up, whereas volume loss represents the complementary reduction from the initial postoperative volume. The height (projection) and width of the reconstructed nipples were measured using a ruler and photographed before, immediately after operation, and during each outpatient visit (2 weeks, 1 month, 3 months, 6 months Post-Op) (Figure 2, Figure 3 and Figure 4). All measurements were performed by the same physician to ensure consistency. The rate of reduction in the projection and volume retention rate (or persistence rate) were calculated as follows:

Volume retention rate (Persistence rate) (%) = 100 × [π × height × (1/2 diameter)^2^ at the last measurement/π × height × (1/2 diameter)^2^ immediately after surgery]

Volume loss rate (%) = 100 − Volume retention rate (%)

The losses in projection calculated using this method were statistically compared using STATA (StataCorp. 2021. Stata Statistical Software: Release 17. College Station, TX, USA: StataCorpLLC.), The normality of data distribution was assessed using the D’Agostino-Pearson normality test. The long-term projection losses in nipples reconstructed with the long V-Y flap were compared with those reconstructed using other flaps [7,11,12,13] using an independent two-sample *t*-test. A two-sided value of *p* < 0.05 was considered statistically significant in all analyses.

In their thesis on nipple reconstruction using angel flaps, based on a literature review, Wong et al. compiled and organized the results of nipple reconstruction using 15 different flaps, and we selected the data with relatively clear results [11]. Data reported by Shestak et al. (skate flap, modified star flap, and bell flap), Kroll and Hamilton (double opposing tab flap), and Rubino et al. (modified star flap, arrow flap) were used as controls [7,12,13]. Since Kroll and Hamilton’s data did not include the standard deviation (SD) for projection loss; for our analysis, we assumed that the SD was equivalent to that observed in our study. This assumption was necessary to calculate the *p*-value and compare the projection loss between their study and ours [12].

## 3. Results

A total of 27 patients were compared and analyzed. They were followed up for an average of 6.4 months. The average height and width of the nipples reconstructed with long V-Y flaps were 8.19 mm and 10.85 mm immediately after the operation and 5.33 mm and 10.67 mm six months later, respectively (Figure 2, Figure 3 and Figure 4). The average reduction in projection was 32.79% (95% CI, 27.36–38.22), and the average volume loss was 34.23% (95% CI, 28.13–40.33). Hence, the persistence rate of volume was 65.77%.

A literature review revealed that most studies had data only for projection loss and no data for volume loss [7,11,12,13]; hence, we could only compare the projection loss. Accordingly, comparisons with previously published techniques were performed for exploratory purposes only and should be interpreted with caution. Table 2 presents a comparative analysis of nipple projection loss observed with various flap techniques, illustrating the differential outcomes associated with each method.

For these exploratory comparisons, historical mean values were compared with those of our cohort under the assumption of comparable measurement methods, and standard deviations from our dataset were applied when not reported in the original studies. The projection loss for nipples reconstructed using the long V-Y flap appeared numerically lower (32.79%) than for those that used the skate flap (40.11%, *p* = 0.473) and the modified star flap (40.28%, *p* = 0.415), although these differences were not statistically significant [7].

In contrast, greater projection loss has been reported for the bell flap (69.95%, *p* < 0.001) [7], double opposing tab flap at 10 months (66.00%, *p* < 0.001), modified star flap (69.90%, *p* < 0.001), and arrow flap (50.90%, *p* = 0.020) [12,13,14,15,16,17,18]. While exploratory statistical comparisons yielded *p*-values < 0.05 for these techniques (*p* < 0.001, *p* < 0.001, *p* < 0.001, and *p* = 0.020, respectively), these findings should be regarded as descriptive trends rather than definitive inferential evidence due to methodological limitations inherent in historical comparisons.

## 4. Discussion

In patients with breast cancer undergoing mastectomy for nipple metastasis, nipple reconstruction is often pursued after satisfactory reconstruction of the breast mound. This step is crucial for enhancing the aesthetic completeness of the reconstructed breast and achieving symmetry with the contralateral unaffected breast. Composite grafting involves the transfer of tissue from the healthy nipple and typically exhibits a lower volume loss rate compared to flap-based nipple reconstruction. However, this approach necessitates the sacrifice of healthy tissue and carries a risk of graft failure. Consequently, flap surgery has become the more prevalent choice for nipple reconstruction.

In patients undergoing SSM, the limitation of skin availability poses a significant challenge to achieving successful outcomes from flap surgery, as reusing affected skin often leads to suboptimal results. This difficulty has been corroborated in numerous studies. Various flap surgery techniques have been proposed for nipple reconstruction, including the skate, star, C-V, bell, arrow, and double opposing flaps [1,2,3,4,5,6,9,14]. Despite these multiple techniques, achieving optimal results remains challenging. Studies by Banducci et al., Bramhall et al., and others have consistently shown a 25–50% reduction in flap size within 2–3 months post-operation and a 40–70% reduction at extended follow-up [8,15]. Given these limitations, achieving a flap volume retention of 60% is often considered acceptable in clinical practice. Notably, the long V-Y flap technique used in this study demonstrated a volume persistence rate of 65.77%, suggesting potential effectiveness in mitigating postoperative nipple volume loss and supporting its role as a promising option for short- to mid-term outcomes.

**Figure 5:**
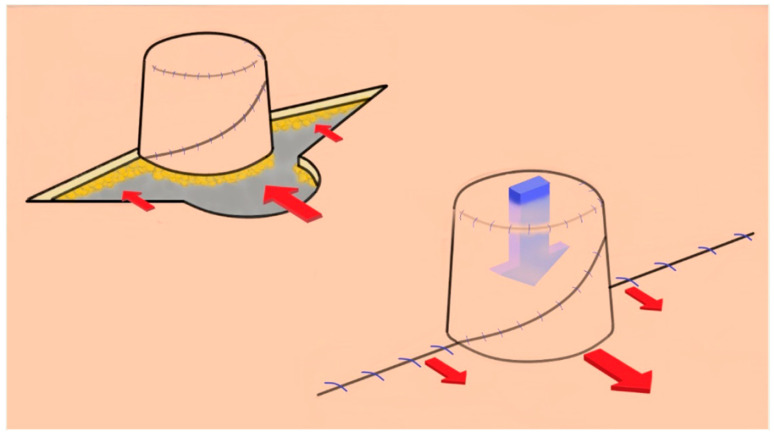
Nipple reconstruction using the CV flap method. Shown is the nipple reconstruction using the CV flap method illustrating various mechanisms that contribute to the reduction in nipple size post-reconstruction. The red arrows indicate the skin retraction forces triggered by the closure of the donor defect, leading to retraction of the adjacent skin. The blue arrow represents two factors: (a) the number of lobules, where multiple lobules increase scar formation and disrupt the blood supply, potentially resulting in necrosis, fat atrophy, and (b) external pressure forces exerted by clothing or posture.

Compensating for this volume loss by initially creating a larger nipple leads to disproportionate sizes often [16]. For example, to achieve a final size of 6 × 6 mm after expected volume loss, a nipple of about 18 × 18 mm must be constructed, i.e., three times the desired size. With techniques like the star or skate flap, experienced surgeons are acutely aware of how such large flaps can distort the overall shape of the breast mound. Consequently, after six months, patients are often left with a mere 6 × 6 mm-sized flap, falling short of the desired esthetic outcomes. This is because many reconstruction techniques fail to maintain the original size, leading to volume loss, as illustrated in Figure 5 [1,7,17,18].

Contributing factors are discussed below:

(1) Retraction force, i.e., tension required to return to the original skin position

In patients undergoing SSM, after tissue expansion processes, there is considerable tension in the skin over the implant. In this condition, when nipples are created through flap surgery, the surrounding skin must be pulled to close the donor defect at the base of the flap, creating retraction force. The larger the flap, the greater the tension.

(2) Number of lobules

Most flaps are designed to have multiple lobules centered on one pedicle to form the cylindrical shape of the nipple. However, with a higher number of lobules in the flap, two problems affected the volume loss rate of the reconstructed nipple. First, based on the number and complexity of the lobules, the number of scars where lobules touch each other increases during the three-dimensional assembly. Second, as many incisions are made, the possibility of disturbing blood flow to the flap increases, leading to fat necrosis and subcutaneous fat atrophy. These factors can act as vectors that reduce volume and pull the nipple downward.

(3) External pressure

External pressure caused by clothing or posture can cause deformation that crushes the reconstructed nipples. Dressings or external devices can adequately protect this but cannot be ruled out permanently. In addition, the absence of a substructure that reinforces the lower part of the reconstructed nipple has been suggested as a cause. Various methods have been proposed using materials such as ear cartilage, ADM, and subcutaneous inlay grafts to reinforce the inner and lower nipples [12,15,19]. However, these methods have limitations owing to additional surgery and donor site morbidity, foreign body reaction, increased operation time, and high cost.

Although it is impossible to eliminate all these factors by changing the design of the flap, the factors mentioned above can be significantly reduced. We used a ‘long V-Y flap’ design to reconstruct the nipple [20]. The V-Y advancement flap is a traditional and effective tissue transfer method used to treat soft tissue defects in the face and other areas. There have been few reports of nipple reconstruction using this method, including a study by Riccio et al., who used a very small V-Y flap for autologous tissue breast reconstruction [21]. They designed the tip of the flap to be located at the base of the nipple [21]. In contrast, the long V-Y flap has an implant located just below it; therefore, it relies on the dermal plexus from the skin pedicle without blood flow from deep tissues.

The long V-Y flap is longer than the conventional random flap, with a width-to-length ratio of 1:2 to 1:2.5. Despite this geometry, clinically significant distal ischemia was not observed, possibly for reasons related to the tissue expansion process commonly performed in SSM, which may induce adaptive microvascular changes resembling delayed phenomena.

When the flap is folded, two sharp rectangular tips formed on top of the nipple. Cutting these rounds is not recommended because the middle part of the flap becomes narrow, constricting the blood flow. Instead, it was manipulated such that the pointed part was bent inside using a 3-point suture (Figure 1).

The ‘long V-Y flap’ has the following advantages. (1) The direct effect of skin tension on the reconstructed nipples is reduced. After the flap donor site is closed, tension is generated at right angles to the scar; however, a flap that moves within the completed nipple is not subjected to this tension. This was evidenced by a lower decrease in the width of the reconstructed papillae, as the tip of the V-Y flap is located in the middle of the donor site when closed, a gradual decrease in tension occurs, reducing the deformation in the breast mound shape. (2) The incision is small, leading to a small scar after assembly. As a result, the change in nipple size from changes in scars, such as contractures on the surface, is reduced. (3) There is only one lobule in the ‘long V-Y flap.’ Hence, theoretically, there would be no major blood flow disturbances compared to flaps with multiple lobules, reducing the possibility of fat necrosis or atrophy.

Despite these advantages, the long V-Y flap is not immune to deformations arising from external forces, which work in the same manner as in a conventional flap.

To determine whether this theoretical hypothesis translates into a long-term clinical result, we compared nipple size reduction from the long V-Y vs. other flap procedures. Patients were followed up during outpatient visits for an average of six months or longer. The reconstructed nipples were measured and photographed, and the reduction in the projection and volume were calculated. Since most literature focuses on projection loss, this study emphasizes projection comparisons using the long V-Y vs. other flap procedures, and the volume change was used as an additional reference. In line with our hypothesis, the width of the nipple remained stable while height decreased (Figure 2, Figure 3 and Figure 4).

Published literature reports projection reductions ranging from approximately 40% to over 70% [7,8,9,11,12,13], consistent with our prior experience using modified star and C-V flaps. In the present cohort, the long V-Y flap demonstrated a projection reduction rate of 32.79%, suggesting improved resistance to size reduction compared with several conventional techniques.

This study has several limitations that should be acknowledged. First, the study population was highly selective and relatively small, comprising 27 patients from a single institution. Cases with flap necrosis, postoperative complications, and patients with comorbidities such as diabetes mellitus or vascular disease were excluded, despite these conditions being common in the breast reconstruction population. This selection bias may have resulted in more favorable outcomes and limits the generalizability of the findings to routine clinical practice.

Second, the retrospective single-cohort design is inherently subjected to selection bias and unmeasured confounding factors, and the absence of a direct comparator group precludes definitive conclusions regarding the relative performance of the long V-Y flap compared with other nipple reconstruction techniques. Comparisons with previously published techniques were therefore based on heterogeneous historical controls with differing patient populations, follow-up durations, and measurement methods, and should be interpreted as descriptive and exploratory rather than inferential.

Third, patient-reported outcomes, including perceived symmetry, aesthetic satisfaction, and quality of life, were not assessed, despite their increasing importance in the contemporary nipple–areola complex reconstruction literature. Fourth, outcome assessment was limited by methodological constraints, as nipple projection and volume were measured using clinical photographs and ruler-based assessments only, without three-dimensional imaging or objective volumetric analysis. In addition, follow-up duration was heterogeneous across patients (mean 6.4 ± 5.7 months), raising uncertainty as to whether the reported “six-month” projection measurements reflect a uniform postoperative time point. Given the relatively short follow-up period, the findings should be interpreted as reflecting short- to mid-term outcomes rather than definitive long-term results.

Finally, although the sample size was considered adequate for exploratory analysis, a formal power analysis with clearly defined effect size assumptions and a prespecified primary endpoint was not performed. Accordingly, the statistical findings should be interpreted with caution. Future prospective studies incorporating standardized follow-up intervals, direct comparator groups, objective volumetric assessment, and patient-reported outcome measures are needed to further validate these findings.

Anatomical differences in nipple morphology across populations further affect generalizability. Western women tend to have smaller, wider nipples, with an average reconstructed projection of approximately 3.8 mm and a width of 13 mm [9]. In contrast, we achieved an average reconstructed nipple size of 10 mm in width and 8 mm in height, approximately 20% greater in volume than a nipple measuring 4 mm in height and 13 mm in width. Creating such a large nipple with conventional flaps remains challenging; however, the long V-Y flap demonstrated consistent results. Notably, the average width of the nipples reconstructed with the long V-Y flap was 10.85 mm, and the reduction in width over time was minimal (1.66%), resulting in a high width retention rate of 98.34%.

Kroll and Hamilton emphasized that flap width is a critical factor for preserving long-term projection because wider flaps enhance blood supply and reduce complications such as fat necrosis [12]. Based on these findings, the long V-Y flap technique appears to be a suitable and effective option for reconstructing large nipples while maintaining long-term projection and volume.

The ‘long V-Y flap’ technique has a shorter operation time due to the simple procedure. Additionally, the base and direction of the flap can be set arbitrarily, allowing the incorporation of the existing scar, leaving only one Y-shaped scar. In addition, the larger the reconstructed nipple, the greater the effect of pulling the anterior skin of the breast, resulting in a flattening deformation. Long V-Y flap surgery may help reduce this deformation, which is often overlooked because they are considered unimportant or unavoidable during nipple reconstruction.

## 5. Conclusions

In patients undergoing skin-sparing mastectomy, nipple reconstruction using flap techniques tends to result in progressive volume loss, making maintenance of initial projection and volume challenging. In this context, the long V-Y flap technique appears to offer a practical and efficient approach that may help mitigate volume loss, particularly in the reconstruction of relatively large nipples.

Further prospective studies with standardized and longer follow-up periods are needed to more clearly define the durability, safety profile, and potential complications associated with the long V-Y flap technique. Comparative studies with larger sample sizes, direct comparator groups, and diverse patient populations could provide more comprehensive insights into the versatility and adaptability of this technique. Additionally, future research exploring the integration of new materials or innovative surgical approaches in conjunction with a long V-Y flap could open new avenues for enhancing aesthetic and functional outcomes. Finally, systematic evaluation of patient-reported outcomes, including aesthetic satisfaction, symmetry, and quality of life, will be essential to fully assess the clinical value of nipple reconstruction techniques and to guide continued improvement of postmastectomy breast reconstruction.

## Figures and Tables

**Table 1:** Patient demographics and characteristics.

	Value
No. of patients	27
Method of reconstruction	
DTI	0
TEI	27
Mean age ± SD, year	48.5 ± 7.9
Mean BMI ± SD, kg/m^2^	22.6 ± 3.0
Mean follow-up ± SD, month	6.4 ± 5.7
Radiotherapy history	1
Chemotherapy history	1
Mean immediate reconstruction nipple height ± SD, mm	8.19 ± 2.35 (95% CI, 7.25–9.12)
Mean immediate reconstruction nipple width ± SD, mm	10.85 ± 1.90 (95% CI, 10.10–11.60)

**Table 2:** Comparison of nipple projection loss rates between different flaps at the six-month follow-up.

	Long V-Y	Skate	Modified Star	Bell
	(N = 27)	(N = 23)	(N = 28)	(N = 17)
Nipple projection loss (%) (standard deviation)	32.79 (13.73)	32.96 (12.70)	29.92 (12.68)	62.64 (28.79)

Confidence intervals were not available for historical controls. No correction for multiple comparisons was performed; therefore, reported *p*-values should be interpreted with caution and considered exploratory rather than confirmatory.

## Data Availability

The data presented in this study are available upon request from the corresponding author. The data are not publicly available due to privacy restrictions.
